# Supplementary material for: Androgen-regulated transcription of ESRP2 drives alternative splicing patterns in prostate cancer
Source: eLife. 2019 Sep 3;8:e47678. doi: 10.7554/eLife.47678 (PMC6788855; doi:10.7554/eLife.47678)
Supplement: Figure 5—source data 1. [file elife-47678-fig5-data1.docx]

| Gene, exon and control | Splicing inclusion in prostate | Protein Function | Function of splice | References |
| --- | --- | --- | --- | --- |
| *ADAM15*  exon 20  ESRP2 activates inclusion | More splicing inclusion in tumour (poorer prognosis isoform) | transmembrane glycoprotein involved in cell adhesion. Over-expressed in prostate cancer, and downregulation promotes metastasis in prostate cancer models. | *ADAM15* exon 20 coding information may affect interactions with SH3 domain proteins | (Burdelski et al., 2017; Kleino, Ortiz, Yritys, Huovila, & Saksela, 2009; Kuefer et al., 2006; Najy, Day, & Day, 2008) |
| *MINK1*  exon 18  ESRP2 activates  inclusion | More splicing inclusion in tumour (poorer prognosis isoform) | serine/threonine kinase |  |  |
| *MLPH*  ESRP2 represses exon 9 | Not differentially expressed in tumours, but exon 9 skipping reduced time to biochemical recurrence | Rab effector protein |  |  |
| *MYH10* ESRP2 represses exon 6 | Not differentially expressed in tumours, but exon 6 skipping reduced time to biochemical recurrence | Non-muscle actin dependent myosin |  |  |
| *MYO1B*  ESRP2 represses exon 23* ( exon 23 activated by SRSF1) | Not differentially expressed in tumours, but exon 23 skipping reduced time to biochemical recurrence | Motor protein | Full length isoform is more oncogenic in gliomas, | (Zhou et al., 2019) |
| NUMB  exon 6  ESRP2 activates inclusion | More skipping in tumour (better prognosis isoform – ESRP2 activates the poorer prognosis isoform) | Tumour suppressor that inhibits notch signalling and binds to MDM2 | *NUMB* exon 3 peptide coding information inhibits MDM2 interaction and prevents p53 degradation | (Colaluca et al., 2018) |
| RPS24, ESRP2 represses exon 5 | More skipping in tumour (poorer prognosis isoform) | *RPS24* gene needed for cell proliferation, encodes ribosomal protein | Introduces translational stop so may induce mRNA instability | (Wang et al., 2015) |
| *TUFT1*  ESRP2 activates exon 2 inclusion | Not differentially expressed in tumours, but exon 2 splicing reduced time to biochemical recurrence | Involved with adaptation to hypoxia, mesenchymal stem cell function, and neurotrophin nerve growth factor mediated neuronal differentiation |  |  |

Figure 5 – Source Data 1

Burdelski, C., Fitzner, M., Hube-Magg, C., Kluth, M., Heumann, A., Simon, R., . . . Wilczak, W. (2017). Overexpression of the A Disintegrin and Metalloproteinase ADAM15 is linked to a Small but Highly Aggressive Subset of Prostate Cancers. *Neoplasia, 19*(4), 279-287. doi:10.1016/j.neo.2017.01.005

Colaluca, I. N., Basile, A., Freiburger, L., D'Uva, V., Disalvatore, D., Vecchi, M., . . . Di Fiore, P. P. (2018). A Numb-Mdm2 fuzzy complex reveals an isoform-specific involvement of Numb in breast cancer. *J Cell Biol, 217*(2), 745-762. doi:10.1083/jcb.201709092

Kleino, I., Ortiz, R. M., Yritys, M., Huovila, A. P., & Saksela, K. (2009). Alternative splicing of ADAM15 regulates its interactions with cellular SH3 proteins. *J Cell Biochem, 108*(4), 877-885. doi:10.1002/jcb.22317

Kuefer, R., Day, K. C., Kleer, C. G., Sabel, M. S., Hofer, M. D., Varambally, S., . . . Day, M. L. (2006). ADAM15 disintegrin is associated with aggressive prostate and breast cancer disease. *Neoplasia, 8*(4), 319-329. doi:10.1593/neo.05682

Najy, A. J., Day, K. C., & Day, M. L. (2008). ADAM15 supports prostate cancer metastasis by modulating tumor cell-endothelial cell interaction. *Cancer Res, 68*(4), 1092-1099. doi:10.1158/0008-5472.CAN-07-2432

Wang, Y., Sui, J., Li, X., Cao, F., He, J., Yang, B., . . . Pu, Y. D. (2015). RPS24 knockdown inhibits colorectal cancer cell migration and proliferation in vitro. *Gene, 571*(2), 286-291. doi:10.1016/j.gene.2015.06.084

Zhou, X., Wang, R., Li, X., Yu, L., Hua, D., Sun, C., . . . Yu, S. (2019). Splicing factor SRSF1 promotes gliomagenesis via oncogenic splice-switching of MYO1B. *J Clin Invest, 129*(2), 676-693. doi:10.1172/JCI120279
