# Supplementary material for: Androgen-regulated transcription of ESRP2 drives alternative splicing patterns in prostate cancer
Source: eLife. 2019 Sep 3;8:e47678. doi: 10.7554/eLife.47678 (PMC6788855; doi:10.7554/eLife.47678)
Supplement: Figure 5—source data 2. [file elife-47678-fig5-data2.docx]

| Gene, exon and control | Splicing inclusion in prostate | | Protein Function | Function of splice | References |
| --- | --- | --- | --- | --- | --- |
| *ARFGAP2*  ESRP2 activates exon 6  inclusion | More splicing inclusion in tumour | | ADP Ribosylation Factor GTPase Activating Protein 2 |  |  |
| *DOCK7*  exon 21  ESRP2 represses inclusion | More skipping in tumour | | guanine nucleotide exchange factor for RAC1 and RAC3 Rho small GTPases |  | (Murray et al., 2014) |
| *ENAH*  ESRP2 activates exon 11A  inclusion | More splicing inclusion in tumour | | ENAH controls actin nucleation and polymerisation, modulating cell motility and adhesion | ENAH exon 11A inclusion produces an isoform called MENAα prognostic for patient survival in lung cancer | (Bria et al., 2014; Urbanski, Leclair, & Anczukow, 2018) |
| *EPN3* ESRP2 activates exon 4  inclusion | Not differentially spliced in tumours | | Epsin3 has roles in apoptosis and EMT | Exon 4 includes peptide information for UIMC domain | (Mori et al., 2017; Wang et al., 2018) |
| *FN1* ESRP2 represses exon 25  inclusion | Not differentially expressed in tumours | | Involved in cell adhesion and maintenance of cell shape |  |  |
| *FNIP1*  ESRP2 activates exon 7 inclusion (also controlled by MBLN and RBFOX) | More splicing inclusion in tumour | | Binds to tumour suppressor folliculin and HSP90. Modulates the AMPK and target of rapamycin signaling pathways |  | (Hasumi et al., 2015; Sager, Woodford, & Mollapour, 2018; Venables et al., 2013) |
| *GRHL1*  ESRP2 activates exon 5  inclusion | Not differentially expressed in tumours | | Transcription factor important for iPSCs | Exon 5 inclusion required for GRHL1 protein function as a transcription factor | (Cieply et al., 2016) |
| *KIF13A*  exon 25  ESRP2 represses  Inclusion  (exon activated by MBLN1) | More skipping in tumour | | Microtubule motor that positions endosomes |  | (Venables et al., 2013) |
| RALGPS2  esrp2 activates exon 15 | More splicing inclusion in tumour | | Ras-specific guanine nucleotide-releasing factor |  | (A, Parrini, & Camonis, 2016) |
| *SLC37A2* ESRP2 activates exon 18 | Not differentially spliced in tumour versus normal | Sugar phosphate exchanger 2 | |  |  |
| TCIRG  ESRP2 represses exon 19 | Not differentially spliced in tumour versus normal | T cell immune regulator 1, ATPase H+ transporting V0 subunit a3 | |  |  |

Figure 5 – Source Data 3

A, O. S., Parrini, M. C., & Camonis, J. (2016). RalGPS2 Is Essential for Survival and Cell Cycle Progression of Lung Cancer Cells Independently of Its Established Substrates Ral GTPases. *PLoS One, 11*(5), e0154840. doi:10.1371/journal.pone.0154840

Bria, E., Di Modugno, F., Sperduti, I., Iapicca, P., Visca, P., Alessandrini, G., . . . Nistico, P. (2014). Prognostic impact of alternative splicing-derived hMENA isoforms in resected, node-negative, non-small-cell lung cancer. *Oncotarget, 5*(22), 11054-11063. doi:10.18632/oncotarget.2609

Cieply, B., Park, J. W., Nakauka-Ddamba, A., Bebee, T. W., Guo, Y., Shang, X., . . . Carstens, R. P. (2016). Multiphasic and Dynamic Changes in Alternative Splicing during Induction of Pluripotency Are Coordinated by Numerous RNA-Binding Proteins. *Cell Rep, 15*(2), 247-255. doi:10.1016/j.celrep.2016.03.025

Hasumi, H., Baba, M., Hasumi, Y., Lang, M., Huang, Y., Oh, H. F., . . . Schmidt, L. S. (2015). Folliculin-interacting proteins Fnip1 and Fnip2 play critical roles in kidney tumor suppression in cooperation with Flcn. *Proc Natl Acad Sci U S A, 112*(13), E1624-1631. doi:10.1073/pnas.1419502112

Mori, J., Tanikawa, C., Ohnishi, N., Funauchi, Y., Toyoshima, O., Ueda, K., & Matsuda, K. (2017). EPSIN 3, A Novel p53 Target, Regulates the Apoptotic Pathway and Gastric Carcinogenesis. *Neoplasia, 19*(3), 185-195. doi:10.1016/j.neo.2016.12.010

Murray, D. W., Didier, S., Chan, A., Paulino, V., Van Aelst, L., Ruggieri, R., . . . Symons, M. (2014). Guanine nucleotide exchange factor Dock7 mediates HGF-induced glioblastoma cell invasion via Rac activation. *Br J Cancer, 110*(5), 1307-1315. doi:10.1038/bjc.2014.39

Sager, R. A., Woodford, M. R., & Mollapour, M. (2018). The mTOR Independent Function of Tsc1 and FNIPs. *Trends Biochem Sci, 43*(12), 935-937. doi:10.1016/j.tibs.2018.09.018

Urbanski, L. M., Leclair, N., & Anczukow, O. (2018). Alternative-splicing defects in cancer: Splicing regulators and their downstream targets, guiding the way to novel cancer therapeutics. *Wiley Interdiscip Rev RNA, 9*(4), e1476. doi:10.1002/wrna.1476

Venables, J. P., Lapasset, L., Gadea, G., Fort, P., Klinck, R., Irimia, M., . . . Tazi, J. (2013). MBNL1 and RBFOX2 cooperate to establish a splicing programme involved in pluripotent stem cell differentiation. *Nat Commun, 4*, 2480. doi:10.1038/ncomms3480

Wang, Y., Song, W., Kan, P., Huang, C., Ma, Z., Wu, Q., . . . Zhang, B. (2018). Overexpression of Epsin 3 enhances migration and invasion of glioma cells by inducing epithelialmesenchymal transition. *Oncol Rep, 40*(5), 3049-3059. doi:10.3892/or.2018.6691
