# Supplementary material for: Androgen-regulated transcription of ESRP2 drives alternative splicing patterns in prostate cancer
Source: eLife. 2019 Sep 3;8:e47678. doi: 10.7554/eLife.47678 (PMC6788855; doi:10.7554/eLife.47678)
Supplement: Figure 5—source data 3. [file elife-47678-fig5-data3.docx]

| Gene, exon and control | Splicing inclusion in prostate | Protein Function | Function of splice | References |
| --- | --- | --- | --- | --- |
| CTTND1  ESRP2 represses exon 2 and 3 inclusion | Not differentially spliced in tumours, but exon 2 and 3 splicing reduced time to biochemical recurrence | May activate WNT signalling through binding transcriptional repressor ZBTB33, and may bind and regulate adhesion properties of cadherins. |  | (Schackmann, Tenhagen, van de Ven, & Derksen, 2013) |
| *FLNB*  ESRP2 activates exon 30 inclusion  (also QKI and RBFOX-regulated) | Not differentially spliced in tumour versus normal (but exon 30 included form better prognosis) | Connects cell membrane to actin cytoskeleton and promotes orthogonal branching of actin filaments | Skipping of exon 30 removes H1 domain from FLNB protein, and is correlated with EMT by releasing the FOXC1 transcription factor | (Feng & Walsh, 2004; Li et al., 2018) |
| *ITGA6*  ESRP2 activates exon 25 | More skipping in tumour (exon 25 skipped isoform poorer prognosis) | Membrane protein that functions in signalling and adhesion | ITGA6 splice isoforms encode different C termini proteins that regulate different intracellular signalling pathways | (Groulx et al., 2014) |
| *MAP3K7*  ESRP2 activates exon 12 inclusion | More splicing inclusion in tumour (better prognosis isoform) | Serine/threonine protein kinase that mediates TGFβ and BMP signalling, and controls transcription and apoptosis. *MAP3K7* deleted in 30-40% of prostate tumours, and is associated with a poor clinical prognosis. | Full length Map3K7 promotes TGFβ-mediated apoptosis. Map3k12Δexon12 isoform supports EMT and is expressed in metastatic cell lines | (Goodall et al., 2016; Kluth et al., 2013; Liu et al., 2007; Tripathi, Shin, Stuelten, & Zhang, 2019; Wu et al., 2012) |
| *RAC1*  ESRP2 activates exon 3b (also regulated by hnRNPA1 and SRPKs) | More splicing inclusion in tumour (better prognosis isoform) | RAS superfamily small GTPase | Exon 3b skipping promotes cell mobility | (Wang et al., 2017) |
| SLK  ESRP2 activates exon 13  (also regulated by RBFOX2) | More exon 13 splicing inclusion in tumour (better prognosis isoform) | STE20-like serine/threonine-protein kinase | Exon 30 encodes part of the PKK (polo kinase kinase) domain | (Braeutigam et al., 2014) |
| TRIP10 ESRP2 activates exon 10 | Not differentially spliced in tumour versus normal (but exon 10 spliced isoform better prognosis) | thyroid hormone receptor interactor 10  controls actin cytoskeleton Role in cancer metastasis | Exon 10 within CDC42 interacting domain | (Truesdell et al., 2015) |

Figure 5 – Source Data 2

Braeutigam, C., Rago, L., Rolke, A., Waldmeier, L., Christofori, G., & Winter, J. (2014). The RNA-binding protein Rbfox2: an essential regulator of EMT-driven alternative splicing and a mediator of cellular invasion. *Oncogene, 33*(9), 1082-1092. doi:10.1038/onc.2013.50

Feng, Y., & Walsh, C. A. (2004). The many faces of filamin: a versatile molecular scaffold for cell motility and signalling. *Nat Cell Biol, 6*(11), 1034-1038. doi:10.1038/ncb1104-1034

Goodall, M. L., Fitzwalter, B. E., Zahedi, S., Wu, M., Rodriguez, D., Mulcahy-Levy, J. M., . . . Thorburn, A. (2016). The Autophagy Machinery Controls Cell Death Switching between Apoptosis and Necroptosis. *Dev Cell, 37*(4), 337-349. doi:10.1016/j.devcel.2016.04.018

Groulx, J. F., Giroux, V., Beausejour, M., Boudjadi, S., Basora, N., Carrier, J. C., & Beaulieu, J. F. (2014). Integrin alpha6A splice variant regulates proliferation and the Wnt/beta-catenin pathway in human colorectal cancer cells. *Carcinogenesis, 35*(6), 1217-1227. doi:10.1093/carcin/bgu006

Kluth, M., Hesse, J., Heinl, A., Krohn, A., Steurer, S., Sirma, H., . . . Minner, S. (2013). Genomic deletion of MAP3K7 at 6q12-22 is associated with early PSA recurrence in prostate cancer and absence of TMPRSS2:ERG fusions. *Mod Pathol, 26*(7), 975-983. doi:10.1038/modpathol.2012.236

Li, J., Choi, P. S., Chaffer, C. L., Labella, K., Hwang, J. H., Giacomelli, A. O., . . . Hahn, W. C. (2018). An alternative splicing switch in FLNB promotes the mesenchymal cell state in human breast cancer. *Elife, 7*. doi:10.7554/eLife.37184

Liu, W., Chang, B. L., Cramer, S., Koty, P. P., Li, T., Sun, J., . . . Xu, J. (2007). Deletion of a small consensus region at 6q15, including the MAP3K7 gene, is significantly associated with high-grade prostate cancers. *Clin Cancer Res, 13*(17), 5028-5033. doi:10.1158/1078-0432.CCR-07-0300

Schackmann, R. C., Tenhagen, M., van de Ven, R. A., & Derksen, P. W. (2013). p120-catenin in cancer - mechanisms, models and opportunities for intervention. *J Cell Sci, 126*(Pt 16), 3515-3525. doi:10.1242/jcs.134411

Tripathi, V., Shin, J. H., Stuelten, C. H., & Zhang, Y. E. (2019). TGF-beta-induced alternative splicing of TAK1 promotes EMT and drug resistance. *Oncogene, 38*(17), 3185-3200. doi:10.1038/s41388-018-0655-8

Truesdell, P., Ahn, J., Chander, H., Meens, J., Watt, K., Yang, X., & Craig, A. W. (2015). CIP4 promotes lung adenocarcinoma metastasis and is associated with poor prognosis. *Oncogene, 34*(27), 3527-3535. doi:10.1038/onc.2014.280

Wang, F., Fu, X., Chen, P., Wu, P., Fan, X., Li, N., . . . Hui, J. (2017). SPSB1-mediated HnRNP A1 ubiquitylation regulates alternative splicing and cell migration in EGF signaling. *Cell Res, 27*(4), 540-558. doi:10.1038/cr.2017.7

Wu, M., Shi, L., Cimic, A., Romero, L., Sui, G., Lees, C. J., . . . Cramer, S. D. (2012). Suppression of Tak1 promotes prostate tumorigenesis. *Cancer Res, 72*(11), 2833-2843. doi:10.1158/0008-5472.CAN-11-2724
